# The dynamics of innovative behavior in sports professionals: An ecological dynamics approach to the serial mediation of motivation and cognitive flexibility in the influence of perceived social support

**DOI:** 10.1371/journal.pone.0352660

**Published:** 2026-06-30

**Authors:** Hasan Buğra Ekinci, Ahmet Yavuz Mallı

**Affiliations:** Faculty of Sport Sciences, Erzincan Binali Yıldırım University, Erzincan, Turkey; Çanakkale Onsekiz Mart University: Canakkale Onsekiz Mart Universitesi, TÜRKIYE

## Abstract

This study aims to explain how perceived social support is associated with innovative behavior in sports professionals. The psychological processes explaining this relationship are not sufficiently explained in the literature; in particular, the roles played by intrinsic motivation and cognitive flexibility in this process need to be investigated in more detail. In this context, the sequential mediating roles of these two variables were tested within the framework of the Ecological Dynamics Approach, and a comprehensive model that could contribute to the development of innovative practices was proposed. This cross-sectional study was conducted using data collected from 243 sports professionals working in the province in Eastern Turkey. To examine the predictive role of perceived social support on innovative behavior, a series of mediation models were tested using the PROCESS macro. In this model, intrinsic motivation and cognitive flexibility were positioned as sequential mediators. The analysis revealed that perceived social support did not have a statistically significant direct link with innovative behavior; instead, this relationship was mediated entirely through intrinsic motivation and cognitive flexibility. Specifically, the sequential mediation pathway was found to be significant, in which perceived social support is positively associated with intrinsic motivation and then cognitive flexibility, which in turn relates to higher levels of innovative behavior. The findings support the Ecological Dynamics Approach, which posits that innovative behavior is a dynamic product of individual-environment interaction. Social support functions not only as a motivational factor but also as a contextual affordance that supports cognitive flexibility. This study provides a theoretical and practical framework for explaining the psychological mechanisms that support innovation in sports.

## Introduction

Innovative behavior is a multidimensional phenomenon that encompasses the process of individuals questioning existing practices, developing new ideas, and putting these ideas into practice. This behavior involves not only the generation of ideas but also the determination to implement them [1]. In today’s rapidly changing and uncertain sports environment, sports professionals are expected to demonstrate not only technical competence but also creative, flexible, and adaptable behavior [2]. This requirement directly affects both individual success and organizational effectiveness.

Considering the multi-layered structure of sports, which is not limited to the moment of competition, the necessity of innovative behaviors becomes even more apparent. Beyond the performance reflected in the field, the organizational and technological infrastructures that enable this performance to emerge are also undergoing constant transformation. For example, the process of organizing a sports match requires extensive planning and coordination, from the field surface to the spectator stands, from scoring systems to broadcasting equipment. Innovations such as camera positioning, real-time data transmission, digital platforms that enhance viewer interaction, and even VAR (Video Assistant Referee) systems that increase the transparency of referee decisions are products of creative solutions in this field. Sports professionals must adopt innovative ways of thinking to ensure that not only the players but every stage of the organization can adapt to evolving technology and audience expectations.

In this context, innovation in sports should not be narrowly viewed as a skill focused solely on on-field athletic performance. Rather, as in different organizational areas such as the public sector [3] or education [4], innovation in sports is a comprehensive competence that actively engages in many dimensions, including logistics, media management, audience experience, and ethical decision-making processes. Therefore, innovative behavior should be evaluated as a strategic competency that makes all components of sports more functional, sustainable, and effective both on and off the field, ultimately directly affecting the organization’s overall performance [5]. For this strategic competency to emerge at the organizational level, it is necessary to understand the psychological and social dynamics that feed it at the individual level.

Innovative behaviors emerge as a joint product of individuals’ intrinsic motivations, cognitive resources, and environmental interactions [6]. In this context, social support emerges as an important psychosocial resource. Social support encompasses the experiences of trust, belonging, and approval that individuals derive from their environment; it strengthens their ability to cope with uncertainty and supports motivational processes [7,8]. In particular, perceived social support (PSS) is a cognitive assessment and belief system regarding the individual’s ability to receive support when needed [9,10]. This belief nourishes hope [11] and mental resilience [12], which the individual uses as psychological resources, especially under stressful and uncertain conditions. As a result of these psychological resources, it has been empirically demonstrated that PSS increases an individual’s sense of self-efficacy [13] and, more importantly, their tendency toward creative thinking and behavior. Indeed, a recent experimental study has shown that perceived social support directly increases creativity [14]. At this point, how the individual perceives environmental support elements and how they transform this perception into behavioral outputs brings to the fore a psychological process centered on the concept of motivation.

Motivation is a critical psychological variable in terms of how individuals perceive environmental support and how they translate this perception into behavior [15]. However, the theoretical precedence of motivation over cognitive flexibility is based on the requirement of an antecedent state that facilitates high-effort cognitive engagement. Specifically, the active restructuring of existing knowledge structures and mental models—which constitutes the core of cognitive flexibility—is a resource-intensive executive process that is contingent upon the mobilization of intentional resources. In this regard, intrinsic motivation provides the necessary psychological basis for the individual to transcend stable cognitive patterns and engage in exploratory mental processes. At this point, the individual’s cognitive flexibility comes into play. Cognitive flexibility is not merely a behavioral trait but also the capacity to actively restructure existing knowledge structures and mental models in light of new information [16,17]. Indeed, the current literature positions cognitive flexibility as a central cognitive mechanism closely related to other executive functions and forming the basis of adaptive learning [18,19]. Individuals with cognitive flexibility have an advantage in developing innovative and functional strategies by interpreting contextual information more effectively. This skill becomes even more decisive in sports contexts where there is time pressure, high competition, and environmental uncertainty. These psychological mechanisms, which highlight an individual’s capacity to adapt to environmental conditions, can be understood more comprehensively within theoretical frameworks that view human behavior not as fixed structures but as a holistic entity shaped by contextual variables. The Ecological Dynamics (ED) approach offers a comprehensive theoretical framework that focuses on multi-level and contextual interactions between the individual and their environment, rather than explaining behavior through linear cause-and-effect relationships [20,21]. This perspective emphasizes an individual’s ability to adapt their behavior based on information from the environment and task requirements, highlighting that expert skills are characterized by functional variability and multiple solution paths rather than fixed patterns [22]. Within this framework, the effective use of environmental feedback is central to performance development [23], as learning environments that represent real-life conditions enhance an individual’s adaptive behavioral repertoire [24]. This capacity for adaptation transcends physical movement; the same principles of functional variability apply to cognitive and strategic domains, where sports professionals must reorganize their mental and professional responses to shifting organizational demands. Grounded in concepts such as affordances (i.e., opportunities for action), constraints, and self-organization, ED seeks to explain the interactions between individuals and their environments through a non-linear and context-sensitive lens [25]. Recently, this holistic perspective has been increasingly applied to broader domains, including organizational behavior, where constructs such as social support are reconceptualized as behavioral affordances that create the necessary conditions for strategic adaptation and innovative behavior [26]. Within this framework, constructs such as social support are reconceptualized—not merely as external resources but as affordances that create conditions for behavioral change, creativity, and strategic adaptation.

The current study builds on this perspective by proposing that perceived social support functions as a behavioral affordance that shapes individuals’ motivational and cognitive dynamics. Specifically, it explores the sequential mediating roles of intrinsic motivation and cognitive flexibility in the relationship between perceived social support and innovative behavior among sports professionals. In doing so, it offers a contextual and dynamic model that addresses existing gaps in the organizational psychology literature, particularly in explaining adaptive and innovative behavior in sport contexts.

### Current study

Although organizational psychology literature has long established a relationship between social-motivational factors and innovative behavior [1], our understanding of the psychological mechanisms underlying this process remains fragmented and inadequate, particularly in the context of high-pressure professional athletes. Two key gaps are evident in the current literature:

First, the underlying mechanism remains insufficiently specified. Although variables such as social support, motivation, and cognitive flexibility have often been examined as independent predictors, the extent to which these elements operate as an integrated and dynamic system and the sequential pathways through which they influence one another has not yet been empirically tested. Thus, the mechanistic pathway explaining how an environmental resource (social support) transforms into a complex behavioral outcome (innovative behavior) remains unclear.

Second, the importance of context has not been sufficiently conceptualized. The modern sports ecosystem expects professionals not only to perform at the highest technical level but also to develop psychological resilience and adaptive responses to constantly changing environmental demands (media, organization, technology) [2]. This complex and dynamic context requires non-linear, interactive psychological models.

Indeed, although experimental evidence suggests that social support can enhance creativity [14], the psychological mechanisms through which this effect unfolds and the relative ordering of these mechanisms remain insufficiently understood.

Aiming to address these theoretical and empirical gaps, the present study draws on the Ecological Dynamics Approach to propose a comprehensive model explaining the effect of perceived social support on innovative behavior. Specifically, it seeks to clarify the underlying process by testing the serial mediating roles of intrinsic motivation and cognitive flexibility in this relationship.

In this context, the hypotheses of the study are as follows:

**Hypothesis 1:** Perceived social support will be positively related to innovative behavior.

This hypothesis draws upon prior research emphasizing that social support functions as a key contextual facilitator for the emergence of innovative behavior. A socially supportive environment has been shown to enhance individuals’ willingness to take risks, bolster psychological resources, and provide a psychologically safe space for idea exploration and experimentation.

Empirical findings highlight the significance of a social climate conducive to innovation [27], as well as the direct effect of perceived social support on creativity [14]. In line with this evidence, the present study hypothesizes that higher levels of perceived social support will be positively associated with greater engagement in innovative behavior.

**Hypothesis 2:** Motivation will play a mediating role in the relationship between perceived social support and innovative behavior.

This hypothesis suggests that part of the effect of social support arises by transforming the individual’s motivational state. The logic of this mediating effect is based on two fundamental steps:

First, social support is expected to increase the individual’s intrinsic motivation. According to the Self-Determination Theory, a supportive environment directly promotes intrinsic motivation by nurturing the individual’s basic psychological needs of relatedness and autonomy [8,15]. Within the Ecological Dynamics framework, social support functions as a socio-contextual affordance that is internalized through the fulfillment of these needs, providing the organismic basis for a persistent motivational drive.

Second, the literature consistently finds that this increased intrinsic motivation serves as a fundamental driving force for innovative behavior. Individuals with high intrinsic motivation are more committed to their tasks, more persistent in the face of challenges, and more inclined to seek creative solutions [28].

**Hypothesis 3:** Cognitive flexibility will play a mediating role in the relationship between perceived social support and innovative behavior.

This hypothesis is grounded in the transformational impact of social support on cognitive functioning. The rationale behind this proposed mediation pathway rests on a two-step cognitive mechanism.

First, social support is theorized to enhance individuals’ cognitive flexibility. A psychologically supportive environment fosters psychological safety and reduces perceived stress, which in turn allows individuals to redirect cognitive resources from defensive processes toward exploratory thinking. When individuals feel psychologically secure, they are more likely to disengage from rigid, habitual thought patterns and adopt a more flexible and open-minded cognitive stance [29,30]. This psychological state acts as a stabilizing contextual constraint that facilitates the system’s self-organization toward exploratory cognitive patterns.

Second, heightened cognitive flexibility is expected to provide the cognitive basis for innovative behavior. Flexible cognitive structures allow individuals to reassess novel or uncertain situations and generate adaptive strategies in response to shifting environmental demands through learning and reflection [18].

Taken together, this two-step mechanism suggests that social support indirectly fosters innovation by first enhancing cognitive flexibility, which in turn enables individuals to engage more readily in innovative behavior.

**Hypothesis 4:** Motivation and cognitive flexibility will explain the relationship between perceived social support and innovative behavior through serial mediation.

This hypothesis posits that the positive influence of perceived social support on innovative behavior, as previously demonstrated in organizational research [31,32], does not follow a direct linear path, but instead unfolds through a multi-step psychological sequence in which one process activates the next.

First, perceived social support is expected to enhance intrinsic motivation, consistent with evidence indicating that environmental affordances can shape core motivational states that underlie human behavior [15].

Second, increased intrinsic motivation is theorized to stimulate cognitive flexibility. Prior research has demonstrated that intrinsically motivated individuals are more likely to engage in exploratory thinking and adaptive cognitive restructuring [33].

Finally, this motivation-induced flexibility is expected to facilitate the generation of novel and adaptive solutions to complex challenges, thereby promoting innovative behavior [18].

Thus, this hypothesis underscores a sequential mediation model, in which social support fosters innovation through a cascading pathway involving motivational and cognitive mechanisms.

## Method

### Research model

The purpose of this study is to examine how perceived social support has a predictive role in innovative behavior among sports professionals. Specifically, the research investigates whether this relationship is sequentially mediated by intrinsic motivation and cognitive flexibility.

To address this aim, a correlational survey design was employed as a quantitative research method. This design seeks to identify the direction and strength of associations between two or more variables [34,35].

Given the inherently complex nature of variable interactions in the social sciences, relying solely on simple or direct associations may yield incomplete explanations. In this context, mediation analysis offers a more nuanced understanding by uncovering the underlying mechanisms that explain how and why variables are related.

Furthermore, it has been emphasized in the literature that testing mediating pathways not only enriches the empirical body of knowledge but also provides distinctive theoretical insights into relational structures [36].

### Research group

The research group for this study consisted of sports professionals operating in a province in the Eastern Anatolia Region of Turkey. This setting was purposefully selected due to its distinctive socio-economic profile. Although this province is not a major metropolitan center, it is a developing city characterized by increasing openness to innovation. In recent years, growing investment in sports, rising youth interest, and the strengthening of institutional sports infrastructure have made this locale a meaningful context for examining innovation-related behaviors. It offers a unique opportunity to investigate how innovative practices emerge within a developing ecosystem—outside the resource-concentrated environments of large cities and beyond the static conditions of less dynamic regions.

Participant selection was based on purposive sampling, a method designed to access individuals who exhibit specific characteristics aligned with the study’s objectives [37]. Access to participants was facilitated through coordination with the relevant provincial directorate of youth and sports and affiliated local sports clubs. While purposive sampling enables a deep contextual analysis, the generalizability of findings to the broader population of sports professionals may be limited.

The required sample size was determined using G*Power 3.1. The power analysis was conducted with an alpha level of 0.05, a statistical power of 0.80, and an expected correlation coefficient of 0.20. Based on these parameters, a minimum of 193 participants was deemed necessary [38]. However, consistent with the literature, increasing the sample size was expected to enhance the reliability of results and reduce estimation error [39]. Ultimately, the study included 243 participants: 96 women (39.5%) and 147 men (60.5%), including 124 sports staff (51%) and 119 coaches (49%), with a mean age of 36.6 years.

In this study, the term “sports professionals” refers to two complementary occupational categories: coaches and sports staff (e.g., administrative managers, facility supervisors). As emphasized in the introduction, this distinction reflects the multifaceted nature of innovation in sports organizations. Coaches directly influence on-field performance by developing technical, tactical, and psychological strategies. Meanwhile, sports staff manage and structure the off-field environment that enables such performance to occur. Examining both groups collectively allows for a more holistic understanding of innovative behavior in sport, encompassing both performance-based (on-field) and organizational (off-field) dynamics.

### Data collection tools

#### Perceived social support scale.

The Multidimensional Scale of Perceived Social Support (MSPSS) measures the extent to which individuals feel supported by their families (e.g., “My family is willing to help me make decisions”), friends (e.g., “My friends try to help me”), and other people they consider important in their lives (e.g., partner, fiancé, relative, neighbor, etc.). The scale consists of a total of 12 items and includes three subscales.

The Turkish adaptation of the scale was carried out by Eker et al. [40]. The construct validity of the Turkish form was tested using principal component factor analysis, and a three-factor structure explaining 75% of the total variance was obtained. The internal consistency coefficients of the scale were reported to range from.80 to.95. In this study, Cronbach’s Alpha coefficient was calculated as.83, indicating that the scale has a high level of internal consistency.

The model fit indices obtained from confirmatory factor analysis (CFA) indicate that the proposed model partially fits the data (χ²/df = 2.79, CFI = .923, TLI = .894, RMSEA = .096, SRMR = .0845). Although the RMSEA value is above the recommended threshold, the acceptable levels of CFI and SRMR values indicate that the model has a generally acceptable fit when evaluated according to multiple criteria [41]. However, the TLI value being slightly below the.90 threshold indicates that the model has areas open to improvement.

As a result of the modification index analysis, the error covariances e3 ↔ e4, e5 ↔ e6, and e11 ↔ e12 were included in the model. Following this intervention, a significant improvement in the model’s fit was observed. These modifications were theoretically justified due to semantic overlaps and similar sentence structures observed between the respective items within the Turkish adaptation of the scale

When all these fit indices are evaluated together, it was concluded that the proposed three-factor structure is partially consistent with the data; however, limited structural adjustments are needed to improve the model’s overall fit.

#### Innovative behavior scale.

The Innovative Behavior Scale was developed by Scott and Bruce [1] and aims to assess individuals’ tendencies to research and apply new ideas, methods, and technologies in the workplace. The scale consists of six items in total, and participants’ innovative behaviors are measured through statements such as “I research new technologies, processes, techniques, and/or product ideas.” The items are answered using a five-point Likert-type rating scale ranging from 1 (strongly disagree) to 5 (strongly agree).

The scale was adapted to the Turkish context by Çalışkan et al. [42], and it was determined that the single-factor structure of the original form was preserved. Exploratory factor analysis (EFA) was applied in the first sample and confirmatory factor analysis (CFA) in the second sample to examine the construct validity. According to the results obtained, the scale explained 69.28% of the total variance, and the reliability coefficients were found to be high (Cronbach’s α = .93 and.91).

As a result of the analysis conducted in this study, the Cronbach’s Alpha reliability coefficient of the Innovative Behaviors Scale was calculated as.89. The model fit indices obtained from the confirmatory factor analysis of the scale indicate that the proposed single-factor structure fits the data quite well: χ²/df = 1.980, CFI = .993, TLI = .986, RMSEA = .064, SRMR = .0206. These values largely correspond to the good fit criteria proposed by Hu and Bentler [41].

As a result of the analysis of modification indices, the error covariances e2 ↔ e6 and e5 ↔ e6 were included in the model. The decision to correlate these error terms was based on wording similarities between the items, reflecting a shared measurement variance. These interventions resulted in significant improvements in the model’s fit indices, and the proposed single-factor structure was maintained.

When all these findings are considered together, it is evident that the Innovative Behaviors Scale possesses both high internal consistency and strong structural validity in the sample group of this study.

#### Adult motivation scale.

In line with the theoretical basis of this study, only the intrinsic motivation sub-dimension was used. Within the context of Self-Determination Theory [8], this approach is based on the assumption that social support will increase intrinsic motivation by supporting the individual’s basic psychological needs (relatedness and autonomy) [15]. The Adult Motivation Scale was developed by Tulunay Ateş and İhtiyaroğlu [43]. It consists of two sub-dimensions: intrinsic and extrinsic motivation. The intrinsic motivation dimension consists of a total of 13 items, and its reliability coefficient is reported as 0.92. In this study, the intrinsic motivation reliability coefficient was calculated as 0.88.

The model fit indices obtained in the confirmatory factor analysis conducted for this sub-dimension show that the proposed structure fits the data at an acceptable level (χ²/df = 2.34, CFI = .921, TLI = .903, RMSEA = .076, SRMR = .052). According to the threshold values proposed by Hu and Bentler [41] and Kline [44], CFI and TLI values above.90 and SRMR values below.08 indicate strong fit, while RMSEA values close to.08 indicate an acceptable level.

Upon examining the modification indices, adding the error covariances e1 ↔ e2, e2 ↔ e3, and e2 ↔ e5 to the model resulted in a significant improvement in fit and strengthened the model’s structural validity. The error terms were allowed to correlate because the respective items utilized highly similar evaluative adjectives, suggesting a high degree of semantic overlap. Accordingly, it was concluded that the single-factor structure is theoretically supported by the measured sample.

#### Cognitive flexibility scale.

In this study, the Cognitive Flexibility Scale developed by Martin and Rubin [45] and adapted into Turkish by Altunkol [46] was used to determine the participants’ levels of cognitive flexibility. The scores obtained from this 12-item scale range from 12 to 72. High scores on the scale indicate that the individual has a high level of cognitive flexibility.

The internal consistency coefficient reported in the original form of the scale is.81. In this study, Cronbach’s Alpha was calculated as.79, indicating that the scale has sufficient internal consistency.

The model fit indices obtained from the confirmatory factor analysis conducted to test construct validity indicate that the proposed single-factor model fits the data at an acceptable level (χ²/df = 2.12, CFI = .936, TLI = .914, RMSEA = .072, SRMR = .045). These findings are consistent with the threshold values proposed by Hu and Bentler [41] and Kline [44] and indicate that the model aligns meaningfully with the data.

As a result of the analysis of modification indices, adding the error covariances e3 ↔ e12, e7 ↔ e9, and e6 ↔ e7 to the model resulted in a significant improvement in model fit, while maintaining the proposed single-factor structure. This adjustment was justified by the linguistic similarity of the items, which pointed toward a common source of residual variance.

### Data analysis

SPSS 25.0 software was used to analyze the research data. Prior to analysis, the data set was examined in terms of outliers, missing data, and normality assumptions (skewness, kurtosis, Z value, and Mahalanobis distance); it was determined that the data were normally distributed and that there were no multicollinearity issues. The factor structures of the scales were tested using confirmatory factor analysis (CFA) via the AMOS 26 program. The serial mediation model was tested using the Hayes PROCESS macro (Model 6). This macro allows for the separate examination of the indirect effects of each mediator variable and simultaneously evaluates the indirect effect passing through two mediators in a serial manner [47]. In the analyses, the non-parametric bootstrap technique was used with 5,000 resampling iterations to determine the significance of direct and indirect effects.

### Ethical considerations

The study was conducted in accordance with the Declaration of Helsinki and approved by the Ethics Committee for Human Research in Health and Sports Sciences of Erzincan Binali Yıldırım University (Protocol code: 05/14, Date: May 30, 2025). Written informed consent was obtained from all participants prior to their involvement. Data collection was carried out between June 3, 2025, and July 3, 2025.

## Results

Table 1 shows the Pearson correlation coefficients, descriptive statistics (X = mean), skewness (Skewness) and kurtosis (Kurtosis) values, and reliability coefficients (Cronbach’s α) for the variables of perceived social support (PSS), intrinsic motivation (IM), cognitive flexibility (CF), and innovative behavior (IB). A positive relationship was found between PSS and IM (r = .453, p < .01) and positive relationships were observed between PSS and CF (r = .388, p < .01) and IB (r = .391, p < .01). Similarly, the IM variable was found to be positively related to CF (r = .544, p < .01) and IB (r = .630, p < .01). Similarly, a positive relationship was observed between CF and IB (r = .484, p < .01). A Cronbach’s Alpha coefficient above 0.70 generally indicates that the measurements are reliable [48]. The Cronbach α coefficients for the PSS scale (α = .83), IM scale (α = .88), CF scale (α = .79), and IB scale (α = .89) were found to be above 0.70. Kalaycı [49] states that if the skewness and kurtosis coefficients are between +1 and −1, the data will exhibit a normal distribution.

**Table 1:** Correlations between variables and descriptive analyses.

Variables	PSS	IM	CF	IB	X	SD	Skewness	Kurtosis	Cronbach
PSS	1	.453**	.388**	.391**	62.67	9.69	.309	−.209	.83
IM		1	.544**	.630**	55.08	5.00	.545	−.604	.88
CF			1	.484**	55.11	5.08	.579	.084	.79
IB				1	22.37	3.92	.500	−.639	.89

**p < 0.01 N = 243; PSS = Perceived Social Support; IM = Intrinsic Motivation; CF = Cognitive Flexibility; IB = Innovative Behavior.

**Fig 1:**
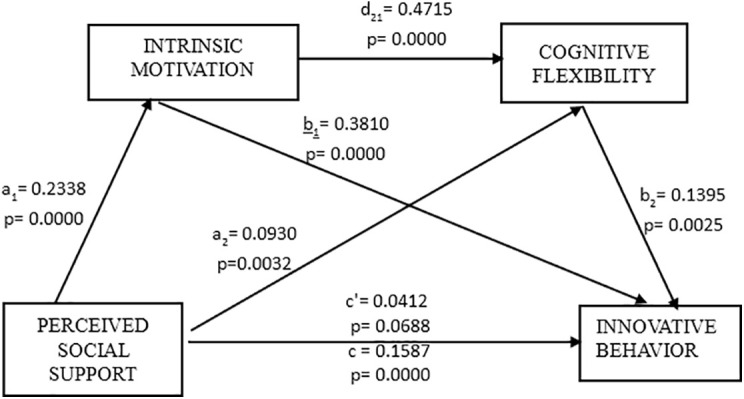
Results of serial mediation analysis.

As presented in Fig 1, perceived social support (PSS) significantly predicted intrinsic motivation (IM) (path a1: β = 0.2338, p < .001) and cognitive flexibility (CF) (path a2: β = 0.0930, p = .0032). Importantly, intrinsic motivation also significantly predicted cognitive flexibility (path d21: β = 0.4715, p < .001), supporting the proposed sequential mechanism underlying the serial mediation structure. In addition, intrinsic motivation (path b1: β = 0.3810, p < .001) and cognitive flexibility (path b2: β = 0.1395, p = .0025) were both significant predictors of innovative behavior (IB). While the direct effect of PSS on IB (c′ = 0.0412, p = .0688) was not statistically significant, the total effect (c = 0.1587, p < .001) was significant, providing empirical support for H1.

As shown in Table 2, the indirect effect of PSS on IB through IM alone was significant (a1b1: β = 0.0891, 95% CI [0.0534, 0.1298]), supporting H2. The indirect effect through CF alone was also significant (a2b2: β = 0.0130, 95% CI [0.0024, 0.0271]), supporting H3. Finally, the serial indirect effect through IM and CF was significant (a1d21b2: β = 0.0154, 95% CI [0.0059, 0.0286]), supporting H4. Overall, the total indirect effect was significant (β = 0.1175, 95% CI [0.0825, 0.1570])

**Table 2:** Analysis results related to the model 6 serial mediation analysis test.

	*Effect*	*BootSE*	*BootLLCI*	*BootULCI*
PSS → IM	*0.2338*	0.0296	0.1754	0.2922
PSS → CF	0.0930	0.0313	0.0314	0.1547
IM → CF	0.4715	0.0606	0.3521	0.5909
IM → IB	0.3810	0.0480	0.2864	0.4755
CF → IB	0.1395	0.0457	0.0495	0.2296
PSS ➔ IM ➔ IB	0.0891	0.0195	0.0534	0.1298
PSS ➔ CF ➔ IB	0.0130	0.0064	0.0024	0.0271
PSS ➔ IM ➔ CF ➔ IB	0.0154	0.00058	0.0059	0.0286
**Direct effect**	0.0412	0.0226	−0.0032	0.0856
**Total indirect effect**	0.1175	0.0191	0.0825	0.1570
**Total effect**	0.1587	0.0240	0.1114	0.2060

PSS = Perceived Social Support; IM = Intrinsic Motivation; CF = Cognitive Flexibility; IB = Innovative Behavior.

## Discussion

This study aimed to explore how perceived social support is associated with innovative behavior among sports professionals, grounded in the dynamic interplay between individual and environmental factors. Interpreted through the contextual and multi-level lens of the Ecological Dynamics Approach (EDA), the findings suggest that innovative behavior is not merely the outcome of stable personal traits but may emerge through interactions between environmental affordances, such as social support, and internal psychological states, namely intrinsic motivation and cognitive flexibility.

Consistent with Hypothesis 1, perceived social support was positively associated with innovative behavior; however, this association did not occur through a direct pathway. Instead, the relationship was primarily reflected through indirect mechanisms, suggesting that social support may operate as a contextual condition that supports innovation when relevant psychological processes are engaged. This interpretation aligns with core EDA principles emphasizing non-linearity, self-organization, and contextual dependency of behavior. Within this framework, social support does not determine behavior on its own, but can be conceptualized as an affordance—a situational opportunity that invites action depending on the individual’s internal state.

Although the serial mediation model (PROCESS macro) introduces a structured pathway, it is conceptualized here as a statistical proxy to approximate cascading interactions within a complex system rather than a deterministic causal chain. From an Ecological Dynamics perspective, the identified sequence from perceived social support to innovative behavior represents an empirically testable approximation of how the person–environment system may self-organize in response to interacting environmental and organismic constraints. In this context, perceived social support may be interpreted as a contextual affordance, whereas intrinsic motivation and cognitive flexibility may be viewed as key organismic constraints shaping the system’s adaptive transition toward an innovative outcome. Nevertheless, linear modeling cannot fully capture reciprocal and time-dependent non-linear dynamics; therefore, future studies may benefit from longitudinal or dynamic modeling approaches to examine emergent processes more directly.

Hypotheses 2 and 3 were supported, as intrinsic motivation and cognitive flexibility both acted as significant mediators. Notably, intrinsic motivation was directly associated with innovative behavior and was also indirectly linked to it through its association with cognitive flexibility, reinforcing evidence that motivational readiness may serve as a prerequisite for adaptive cognitive functioning [33]. The strongest path was the direct link from intrinsic motivation to innovative behavior, highlighting the central role of autonomous drive in professional innovation.

Importantly, the serial mediation model (H4) was statistically significant, indicating a coherent sequential pattern: perceived social support was associated with higher intrinsic motivation, which in turn was associated with greater cognitive flexibility, and this flexibility was further linked to innovative behavior. This pattern illustrates how environmental inputs may be integrated into internal psychological processes and how behavior may emerge through nested interactions across levels. From an EDA standpoint, the results support the assumption that innovative behavior is shaped not by isolated predictors but through co-regulation between contextual affordances and individual adaptability.

Overall, the findings extend existing literature by offering a context-sensitive and system-oriented understanding of innovative behavior in sports organizations. Rather than viewing innovation as a static trait, this study conceptualizes it as an emergent property of dynamic person–environment coupling, supporting the utility of EDA as a theoretical lens in organizational research.

### Interpretation of findings in light of the theoretical framework (ecological dynamics)

#### Social support as an “Environmental Constraint” or “Affordance”.

Within Ecological Dynamics, environmental factors may function as constraints or affordances that limit or expand behavioral possibilities. In this context, perceived social support can be interpreted as a dynamic environmental feature that may be associated with different behavioral tendencies depending on contextual conditions rather than exerting a fixed and unidirectional influence. Empirical studies indicate that the effects of organizational support programs on innovative behavior vary across macro-level contexts such as national culture [50]. Similarly, micro-level social dynamics such as organizational climate [51] or coworker jealousy [52] may act as constraints determining whether support is experienced as an opportunity.

The current findings indicate that the association between social support and innovative behavior is reflected through intermediary mechanisms, supporting its interpretation as an affordance rather than a direct driver. Individuals may experience support not simply as assistance but as an informational resource that may support action, idea development, and strategic adaptation. This interpretation is consistent with evidence linking social support to psychological safety and motivational resources [32,51,53], suggesting that supportive environments may strengthen readiness for exploration.

At the same time, social support may function as a constraint if it creates expectation burdens, reduces autonomy, or conflicts with the individual’s action plans [54]. Thus, whether support is experienced as an opportunity depends on both contextual conditions and the individual’s goals, skills, and perceptual state [55]. This reinforces the EDA argument that social support is dynamic and context-sensitive rather than static.

#### The dynamic role of motivation and cognitive flexibility.

From a dynamical systems perspective, intrinsic motivation and cognitive flexibility represent adaptive regulatory mechanisms that respond to situational demands. The literature indicates that environmental cues may be associated with higher intrinsic motivation, which may subsequently relate to creativity and exploratory behavior across learning and working environments [56,57]. Consistent with this view, our findings show that perceived social support was positively associated with intrinsic motivation, aligning with studies suggesting that supportive climates may relate to creativity through motivational processes [58,59]. This motivational state may translate social information into psychological energy for autonomous behavior and innovation [28].

However, motivational activation alone may be insufficient unless accompanied by cognitive flexibility. Cognitive flexibility reflects the capacity to reorganize strategies in response to changing environmental demands [60] and to maintain a functional balance between stability and flexibility [61,62]. From an ecological perspective, this balance appears to be fundamental for adaptive goal achievement under uncertainty [63,64].

A key contribution of this study is that cognitive flexibility did not operate as a meaningful mediator independently but became influential when linked with intrinsic motivation. This suggests that motivation may serve as a functional precondition enabling cognitive restructuring. In this sense, intrinsic motivation may be interpreted as an initiating mechanism that mobilizes flexibility in ways that support innovative responses.

In conclusion, intrinsic motivation and cognitive flexibility appear to function as interdependent components sensitive to environmental information. Their dynamic interaction may expand behavioral repertoires and support innovative solutions [65], whereas dysfunction in this integrated system may relate to rigid and repetitive behavioral patterns, as observed in neuropsychiatric disorders [66].

#### Serial mediation and “Self-Organization”.

The observed serial mediation structure provides empirical support for self-organization within the person–environment system. Within this framework, innovative behavior is not necessarily the output of a predetermined internal program but may represent an emergent order arising from momentary interactions between internal states and contextual constraints. Innovative behavior can therefore be conceptualized as an adaptive response that may develop under uncertain and variable conditions [67]. Such adaptation cannot be explained solely through individual or environmental factors but through their interaction [68].

In this context, the supported sequence of perceived social support → intrinsic motivation → cognitive flexibility → innovative behavior can be interpreted as an observable trace of self-organization. Internal system components may reorganize in response to environmental inputs, supporting functional behavioral outcomes. These dynamics may contribute to creativity and innovation, potentially supporting sustainable competitive advantage [69]. Accordingly, the model proposed in this study aligns with Ecological Dynamics assumptions regarding contextual and interactive behavioral emergence.

### Dialogue between findings and current literature. Confirmation and expansion:

The findings are consistent with established evidence that social support encourages innovative behavior. This association aligns with experimental findings showing that social support increases creativity [14] and with research emphasizing innovation-supportive climates [27]. Prior studies also suggest that social support influences innovation indirectly by increasing work engagement [70] and organizational commitment [51,71]. These pathways may reflect broader processes through which support fulfills psychological needs and strengthens risk-taking.

Social support may also provide psychological safety that encourages experimentation and tolerance of uncertainty [7]. Similarly, perceived support may be associated with higher self-efficacy and psychological empowerment, thereby supporting proactive and innovative behavior [70,72]. Extending this literature, the present study suggests that empowerment may unfold through a sequential mechanism in which intrinsic motivation is linked to cognitive flexibility, offering a more hierarchical and dynamic alternative to traditional linear models.

***Filling the research gap:*** Although innovative behavior research recognizes the importance of social support, intrinsic motivation, and cognitive flexibility, existing models often remain fragmented and linear. First, the dynamic and hierarchical interactions among these variables have been underexplored, and the question of how social support relates to motivational processes that activate cognitive mechanisms has remained conceptually and empirically insufficiently developed. Second, social support has often been treated as a static resource with unidirectional effects, while the Ecological Dynamics assumption that behavior emerges from momentary person–environment interactions has rarely been integrated into innovation models.

To address these limitations, the present study tested a comprehensive serial mediation model grounded in Ecological Dynamics. By demonstrating that intrinsic motivation may be linked to cognitive flexibility in a sequential pathway, the findings provide evidence for a process that has remained largely overlooked, particularly in sports professionals operating under uncertain and variable performance conditions.

#### Practical implications.

The findings provide an actionable roadmap for managers, coaches, and educators seeking to promote innovation and adaptive performance in sports environments. Effective intervention may benefit from focusing on sequential psychological processes rather than isolated factors.

First, social support may be viewed as a strategic starting point that contributes to psychological safety. Leaders may seek to establish climates in which failure is treated as learning and feedback is not perceived as threat, thereby supporting risk-taking and experimentation.

Second, support may be more impactful when translated into intrinsic motivation. Providing autonomy, involving professionals in designing training processes, and encouraging personal goal-setting may strengthen internal motivational drive.

Finally, motivated individuals may be more likely to mobilize cognitive flexibility. Non-routine problem scenarios, interdisciplinary tasks, and unexpected constraints may disrupt habitual strategies and support innovative solutions. Overall, fostering innovation may be conceptualized as an integrated developmental process rather than a single managerial intervention.

#### Limitations of the study and recommendations for future research.

Despite its contributions, this study has limitations. First, the cross-sectional design restricts causal inference; longitudinal or experimental research is needed to clarify directionality. Second, self-report measures may be influenced by social desirability and response consistency biases; future studies may benefit from mixed-method designs, behavioral observations, or third-party assessments.

Third, the sample was limited to sports professionals from a single province in Eastern Anatolia, Turkey, which may limit generalizability across cultural and institutional contexts. Fourth, although the theoretical framework is grounded in Ecological Dynamics, the analysis relies on quantitative methods; qualitative interviews or situational analyses may provide deeper insight into how affordances are perceived and acted upon.

Finally, the model included only intrinsic motivation and cognitive flexibility as mediators. Future research could incorporate additional mechanisms such as psychological safety, self-efficacy, and creativity climate to provide a more comprehensive explanation. Despite these limitations, the study provides important evidence regarding sequential mechanisms through which perceived social support may be associated with innovative behavior.

## Conclusion

This study presents a multi-level explanatory model emphasizing person–environment interaction to understand innovative behavior among sports professionals. The findings indicate that perceived social support is linked to innovative behavior primarily through a sequential process involving intrinsic motivation and cognitive flexibility. Consistent with Ecological Dynamics assumptions, innovative behavior may be conceptualized as an emergent adaptation arising from the integration of environmental information and internal psychological mechanisms. Overall, the study provides theoretical and practical foundations for multi-dimensional strategies aimed at supporting creative and adaptive behaviors in sports contexts.
